# Charged membrane interfaces reshape the nucleation landscape of RIPK3 amyloid variants

**DOI:** 10.1007/s00249-026-01833-8

**Published:** 2026-03-10

**Authors:** Fátima C. Escobedo-González, Andrea Gelardo, Gustavo A. Titaux-Delgado, Miguel Mompeán

**Affiliations:** 1 Instituto de Química Física Blas Cabrera, Consejo Superior de Investigaciones Científicas (IQF-CSIC), Serrano 119, Madrid 28006, Spain

**Keywords:** Amyloid, NMR, DIBMALPs, DMPG, DMPC, RIPK3

## Abstract

Amyloid assembly is governed by a balance between intrinsic sequence determinants and environmental cues that modulate nucleation. The RIP homotypic interaction motif (RHIM) of receptor-interacting protein kinase 3 (RIPK3) provides a tractable model to dissect these principles. In solution, amyloid formation strictly requires the conserved VQVG RHIM core tetrad; a deliberately core-disrupted variant (VQVG→AAAA) fails to assemble under aggregation permissive conditions. Here, we use Thioflavin-T fluorescence assays and NMR spectroscopy to show that negatively charged lipidic interfaces unlock a latent amyloidogenic potential in this mutant. These results delineate two separable dimensions of amyloidogenesis, namely a sequence-encoded propensity and an environmental component that can catalyze nucleation by templating molecular proximity and orientation.

## Introduction

Receptor-interacting protein kinase 3 (RIPK3) is a central effector of necroptosis, a regulated form of lytic programmed cell death critical for host defense and inflammation (Dhuriya and Sharma 2018). Unlike apoptosis, necroptosis relies on the transient assembly of RIPK3 into functional amyloid fibrils that scaffold the recruitment and phosphorylation of downstream effectors such as mixed lineage kinase domain-like protein (MLKL) (Davies et al. 2024). Once phosphorylated, MLKL oligomerizes at the plasma membrane, disrupting bilayer integrity and triggering cell rupture (Samson and Murphy 2024). While essential for the clearance of infected or damaged cells, dysregulated necroptosis, either excessive or impaired, has been implicated in chronic inflammation, cancer, and neurodegeneration (Ye et al. 2023).

At the molecular level, RIPK3 polymerization is driven by the RIP homotypic interaction motif (RHIM), a conserved amyloidogenic sequence present in multiple necroptotic and innate immune signaling proteins (Li et al. 2012; Wu et al. 2021; Hoblos et al. 2025). Structural studies using solid-state NMR and cryo-electron microscopy have shown that, in the fibrillar state, the RHIM forms a stable amyloid core of parallel β-sheets, while the flanking regions remain flexible and disordered (Wu et al. 2021; Andrés et al. 2025). Biophysical analyses have further revealed that RIPK3 assembly in solution is finely tuned by a balance of electrostatic repulsion and hydrophobic interactions, establishing strict physicochemical conditions under which fibrillization is permitted (Pham et al. 2023; Escobedo et al. 2025). Disruption of the central VQVG tetrad abolishes this assembly, underscoring its indispensable role in nucleation and polymer growth (He et al. 2025).

Beyond bulk solution, increasing evidence indicates that lipid interfaces can profoundly modulate the aggregation of amyloid-forming proteins, including Tau, α-synuclein, and amyloid-β (Frieg et al. 2024; El Mammeri et al. 2025; Sant et al. 2025). For functional amyloids, this concept is only beginning to emerge, as highlighted by recent findings showing that membrane charge can prime RIPK3 for amyloid assembly (Escobedo et al. 2025). Such interfaces may act as active modulators, not only accelerating nucleation by concentrating monomers at the surface but also reshaping the conformational ensemble of the protein. Whether this environmental tuning can expose “cryptic” amyloidogenic regions, that is, segments that are bioinformatically predicted to form amyloid but remain kinetically silent in solution, potentially enabling nucleation through alternative, surface-driven pathways remain unexplored.

Here, we address this question by investigating the effect of a membrane mimetic on the aggregation behavior of a core-disrupted (VQVG→AAAA) RIPK3 mutant. By combining Thioflavin-T fluorescence assays with solution NMR spectroscopy, we reveal an unexpected lipid-driven nucleation pathway in which negatively charged Diisobutylene-Maleic Acid Lipid Particles (DIBMALPs) unlock a latent amyloidogenic potential in the RIPK3 variant, enabling assembly even in the absence of the canonical RHIM core tetrad VQVG. These findings suggest that membrane charge and composition could act as regulatory factors for amyloid aggregation, with implications for the spatial and temporal control of necroptotic signaling in cells.

## Materials and methods

### Protein expression and purification

The human RIPK3 C-terminal domains (CTD; residues 387–518, WT and VQVG◊AAAA variants), codon-optimized for *E. coli* expression, were subcloned into a pET11a-derived vector containing an N-terminal His_6_-tag followed by a TEV protease cleavage site. The construct was expressed in *E. coli* BL21 (DE3) cells. Following the protocol by Sivashanmugam and coworkers (2009), cultures were grown in 2×YT medium at 37 °C until OD_600_ reached 0.6–0.8, harvested, and resuspended in M9 minimal medium supplemented with ^15^NH_4_Cl for isotopic labeling. After 1.5 h of adaptation, protein expression was induced with 0.5 mM IPTG and cells were incubated overnight at 25 °C. Cell pellets were lysed by sonication in buffer (20 mM Tris-HCl, 150 mM NaCl, 1 mM EDTA, pH 8.0), and inclusion bodies were recovered by centrifugation. The insoluble protein was solubilized in 6 M guanidine hydrochloride, 20 mM Tris-HCl, and 2 mM DTT (pH 8.0) and purified under denaturing conditions using Ni^2+^-affinity chromatography. The column was washed with urea-containing buffer and the protein eluted with imidazole. Finally, the eluate was filtered (0.22 μm) and subjected to size-exclusion chromatography (Superose 6 Increase 10/300 GL) to isolate the monomeric protein fraction.

### Preparation of DIBMALPs

DMPC and DMPG lipids (Avanti Polar Lipids) were individually dissolved in phosphate buffer (20 mM Na_2_HPO_4_/NaH_2_PO_4_, pH 6.5) to 20 mM, vortexed, and equilibrated at 30 °C for 15 min. Lipid suspensions were then subjected to nine freeze–thaw cycles and extruded 30 times through a 100 nm polycarbonate membrane to obtain large unilamellar vesicles (LUVs). DIBMA copolymer (Cube Biotech) was reconstituted in the same buffer at 100 mg/mL and added to the LUV suspension at a lipid-to-polymer mass ratio of 1:1. The mixture was incubated at 30 °C for 1 h and then at 4 °C for 16 h to complete DIBMALPs formation. Insoluble aggregates were removed by centrifugation (15,000 × g, 30 min, 4 °C). The supernatant was further purified by size-exclusion chromatography (Superdex 200 Increase 10/300 GL, Cytiva) using an ÄKTA system equilibrated with phosphate buffer (20 mM Na_2_HPO_4_/NaH_2_PO_4_, pH 6.5) and eluted at 0.5 mL/min to remove polymer excess and isolate homogeneous DIBMALP preparations.

### Thioflavin T assays of amyloid assembly

Amyloid formation was monitored under different conditions (pH variation, NaCl, SDS, or DIBMALPs) using Thioflavin T (ThT) fluorescence as reported in LeVine (1993). The assembly buffer contained 1 mM acetic acid, 1 mM TCEP, and 40 µM ThT, adjusted to pH 4.0 or 6.5 with 100 mM Na_2_CO_3_. SEC-purified monomeric CTD-RIPK3 was stored in 8 M urea, 1 mM TCEP, pH 4.0, at 200 µM. For aggregation assays, the protein was diluted to 5 µM by rapidly mixing with assembly buffer to reduce urea to 100 mM. Reactions (100 µL) were set up in black half-area 96-well flat-bottom microplates (Thermo Scientific™ Nunc™, Cat. No. 167008) at room temperature. Fluorescence was recorded immediately after mixing using a POLARstar Omega plate reader (BMG Labtech) with excitation at 440 ± 10 nm and emission at 480 ± 10 nm. Data were collected every 30 s for 20 h, in triplicate, and final protein concentrations were confirmed by A280 using a NanoDrop™ 2000 C spectrophotometer (Thermo Fisher Scientific). ThT traces are reported as the mean of three independent wells (*n* = 3) and variability is shown as standard deviation (SD) as shaded regions.

### NMR experiments

NMR experiments were performed at 298 K on a Bruker Avance Neo 800 MHz spectrometer equipped with a TCI cryoprobe and Z-gradient. Uniformly ^15^N-labeled RIPK3 WT and AAAA constructs were desalted into a 90:10 H_2_O/D_2_O buffer containing 1 mM acetic acid (pH 4.0) and 1 mM TCEP. Protein concentrations were adjusted to ~ 40 µM, as determined by UV absorbance at 280 nm. For membrane-interaction studies, DIBMALPs prepared with neutral (DMPC) or anionic (DMPG) lipids. ^1^H–^15^N HSQC spectra were acquired with 8 scans per increment for RIPK3 WT, RIPK3 mut, and RIPK3 + DMPC DIBMALPs, and 32 scans for both RIPK3 WT and RIPK3 mut in the presence of DMPG DIBMALPs due to lower signal-to-noise caused by the interaction with the lipidic particle. Spectral widths and offsets for all these HSQC experiments were set to 12 ppm and 4.70 ppm for ^1^H, and 20 ppm and 117 ppm for ^15^N, respectively. All spectra were processed using TopSpin 4.4.1 (Bruker Biospin, Germany) and visualized in Poky (Lee et al. 2021).

### ZipperDB and PASTA 2.0 analyses

Amyloidogenic segments were predicted using ZipperDB (https://zipperdb.mbi.ucla.edu/), which evaluates the energetic stability of steric zippers derived from short hexapeptide fragments within an input sequence (Goldschmidt et al. 2010; Rosenberg et al. 2022), and using PASTA 2.0 (http://protein.bio.unipd.it/pasta2/), which estimates residue-wise aggregation free-energy profiles to identify regions prone to form cross-β amyloid-like architectures (Walsh et al. 2014). As input for both predictors, we used the experimentally characterized RIPK3 CTD construct analyzed in this study (residues 387–518) in its WT form and in a variant carrying the VQVG→AAAA substitution within the RHIM tetrad. ZipperDB hits with predicted ΔG values below the default threshold (–23 kcal/mol) were considered to have high amyloidogenic propensity. PASTA 2.0 was run with default parameters, and residue positions falling below the recommended aggregation threshold (-5 PASTA units; 1 PASTA unit = 1.192 kcal/mol) were considered to display elevated amyloidogenic propensity. In both cases, interpretation focused on the RHIM-containing region previously defined to form the amyloid core by solid-state NMR (Wu et al. 2021; Andres et al. 2025).

### AlphaFold3 structural predictions

Structural models for the wild-type and AAAA RIPK3 RHIM segments were generated using AlphaFold3 (Abramson et al. 2024), accessed via the AlphaFold server (https://alphafoldserver.com). The sequences were modeled as octamers, and the predicted local distance difference test (pLDDT) scores were used to assess confidence in backbone geometry and inter-chain orientation, yielding very high (pLDDT > 90) and lower (70 > pLDDT > 50) values for the ordered segments in WT and AAAA sequences, respectively. Molecular graphics and analyses were performed with PyMOL (Schrödinger LLC) for visualization.

## Results

### Bulk solution conditions gate RIPK3 nucleation but not the RHIM tetrad mutant

**Fig. 1:**
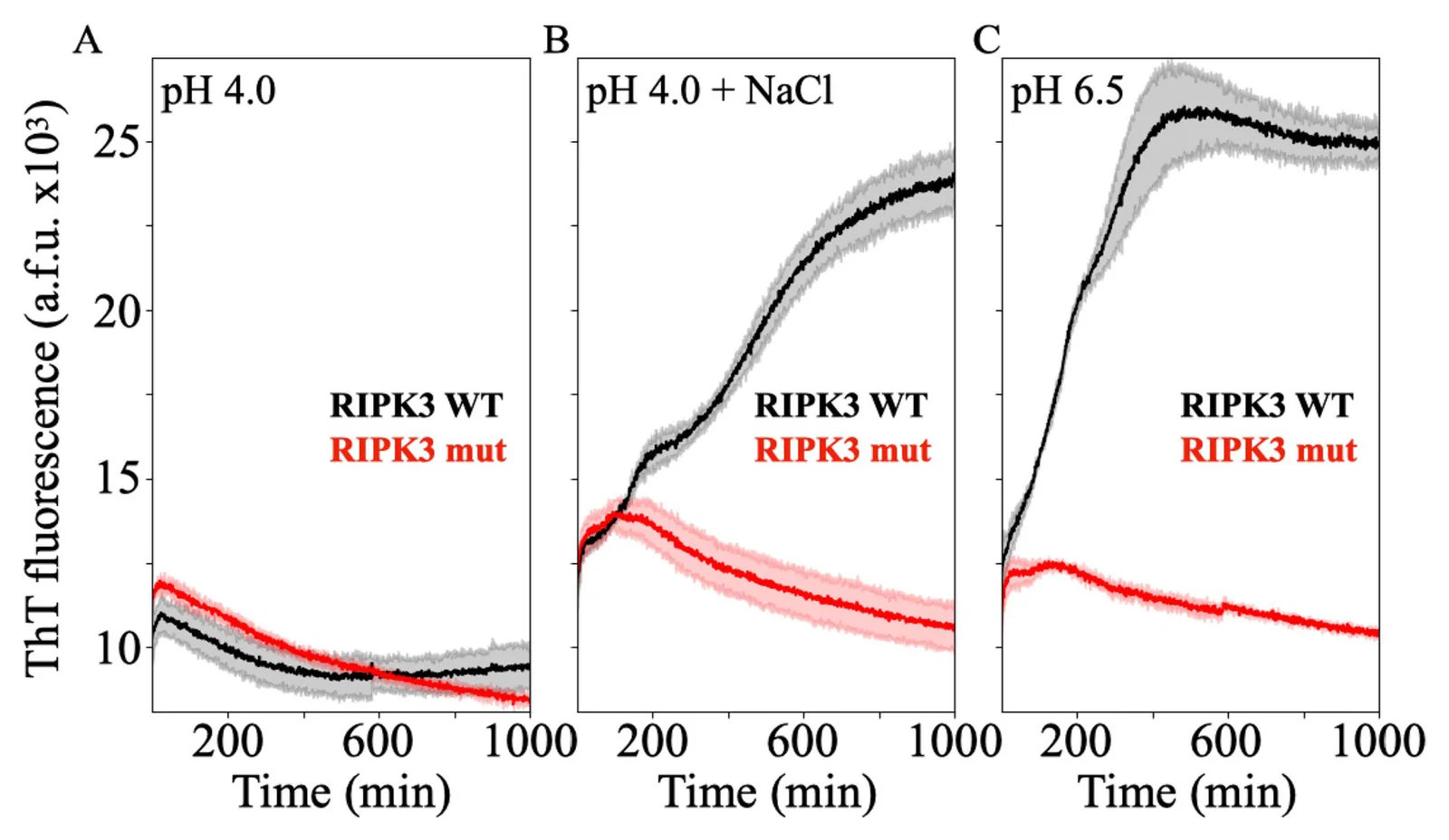
Thioflavin T (ThT) fluorescence assays in bulk solution shows that RIPK3 assembly is strictly dependent on the RHIM core tetrad VQVG. (**a**) At acidic pH (pH 4.0), neither RIPK3 WT nor the AAAA mutant (“RIPK3 mut”) shows detectable ThT-positive nucleation; (**b**) Ionic screening with 150 mM NaCl promotes ThT-positive assembly for WT but not for AAAA variant, indicating that electrostatic modulation alone cannot bypass the compositional requirement of the conserved VQVG tetrad; (**c**) At pH 6.5, WT displays a rapid increase in ThT fluorescence, whereas RIPK3 mut remains at baseline. All ThT measurements are shown as mean with shaded standard deviation from triplicates (three wells for each sample)

Under conditions previously defined as non-permissive for amyloid formation in solution, neither the wild-type (WT) RIPK3 RHIM nor the VQVG→AAAA core mutant showed any detectable signs of aggregation (Fig. 1). At acidic pH (pH 4.0), the ThT fluorescence remained completely flat for both constructs throughout the entire time course, indicating that nucleation and fibril elongation were fully suppressed. This lack of signal is consistent with strong electrostatic repulsion between monomers, which prevents the productive intermolecular associations required for amyloid nucleation, as previously established for WT RIPK3 (Pham et al. 2023; Escobedo et al. 2025). When the ionic strength of the medium was increased by adding 150 mM NaCl, this electrostatic barrier was effectively screened, and the WT protein rapidly transitioned into the aggregated state, as reflected by a robust, time-dependent increase in ThT signal. In sharp contrast, the AAAA variant remained completely inert, with no measurable fluorescence increase even after extended incubation, indicating that screening electrostatics alone is insufficient to drive its nucleation.

When the pH was raised to 6.5, a condition previously shown to favor WT RIPK3 aggregation in solution (Pham et al. 2023; Escobedo et al. 2025), we observed a robust increase in ThT fluorescence, consistent with assembly into amyloids (Fig. 1). Under these near-neutral conditions, the kinetics of the fluorescence rise are indicative of a well-defined nucleation-and-growth process leading to the formation of β-structured assemblies, validating the experimental setup as a reliable baseline to test the behavior of the AAAA RIPK3 variant. Conversely, this variant again showed no detectable aggregation, with its fluorescence baseline remaining flat throughout the experiment, underscoring that disruption of the core tetrad abolishes the intrinsic nucleation capacity of the protein (Fig. 1).

These observations establish two key principles of RIPK3 amyloidogenesis in bulk solution. First, the conserved VQVG tetrad is absolutely required for nucleation: without it, as in the AAAA variant, the protein remains trapped in a monomeric state regardless of electrostatic conditions. Second, electrostatic repulsion acts as an additional gatekeeper, modulating the accessibility of the aggregation pathway for the WT protein but incapable of overriding the intrinsic structural requirement encoded by the RHIM core.

### Bioinformatic scoring supports latent amyloidogenic potential in the tetrad mutant

To evaluate whether the inert behavior of the AAAA variant in bulk solution reflected a true loss of amyloidogenic potential or rather a kinetic barrier to nucleation, we first applied two complementary sequence-based predictors. The RHIM amyloid core previously defined by solid-state NMR spans residues ~ 448–468 and adopts three short β-strands (β1–β3), with the tetrad located in β2 (V458QVG461 in WT; A458AAA461 in the mutant) (Wu et al. 2021; Andrés et al. 2025).

**Fig. 2:**
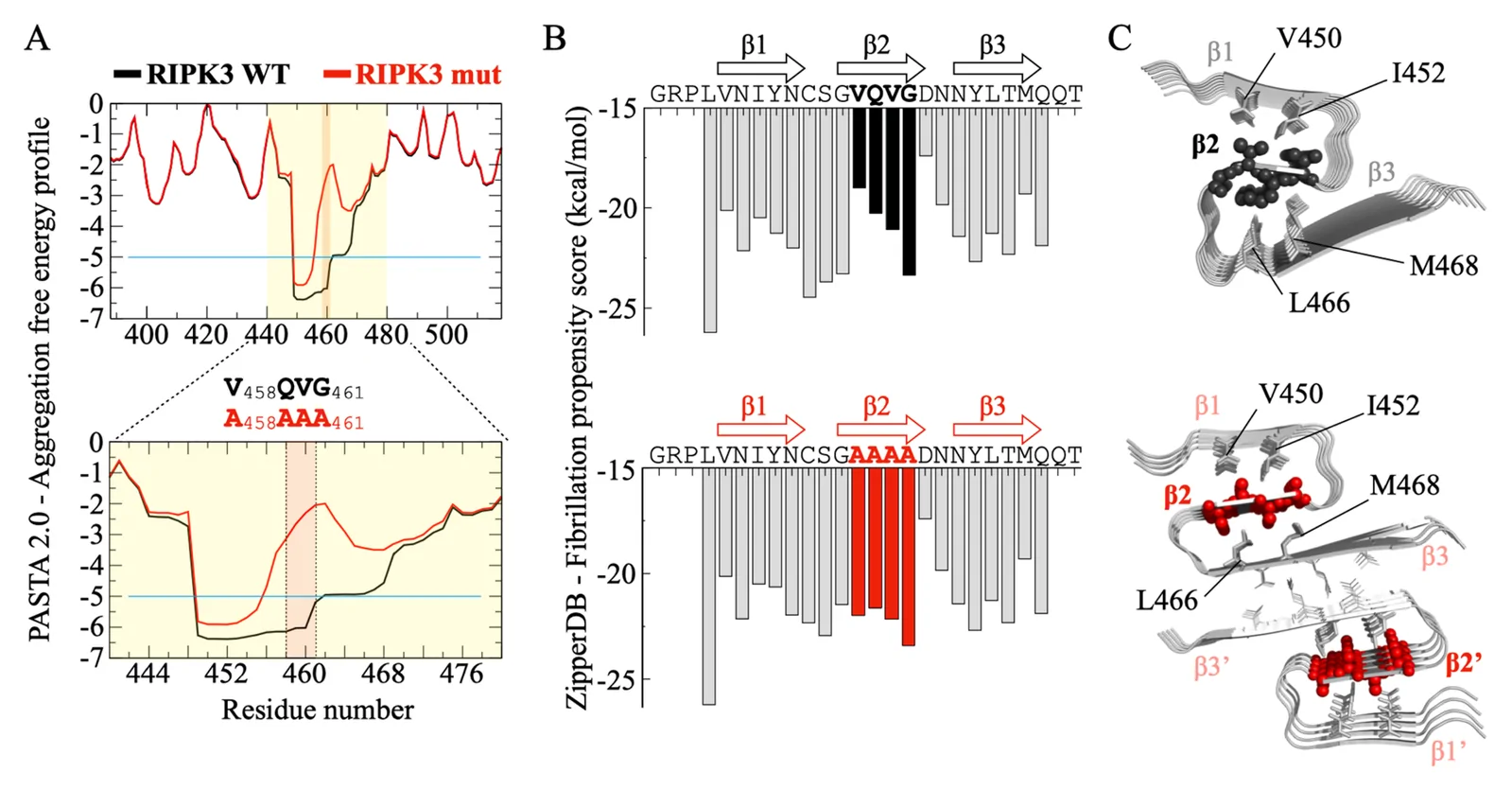
Bioinformatic scoring predicts amyloidogenic potential for both WT and AAAA sequences. (**a**) PASTA2.0 predicts a pronounced aggregation hotspot in WT spanning β1–β2 (residues 449–461, including the VQVG tetrad), whereas in AAAA the profile is attenuated, with the strongest contribution concentrated in β1 and reduced propensity around the mutated tetrad (threshold indicated by a blue line); (**b**) ZipperDB identifies multiple high-scoring hexapeptides across residues 448–470 for both WT and AAAA, including windows encompassing the tetrad region (highlighted); (**c**) AlphaFold models of WT and AAAA RHIM segments; tetrad residues are shown as spheres and neighboring residues contributing to core packing (e.g., L450/I452 in β1 and L466/M468 in β3) are shown as sticks. For AAAA, a dimeric arrangement is predicted; interfacial residues are shown as lines

PASTA 2.0 (Walsh et al. 2014) identified a pronounced aggregation-prone region in the WT sequence spanning the β1–β2 area, including the tetrad-containing window, whereas in the AAAA variant the predicted profile is attenuated and the strongest contribution shifts towards the N-terminal part of the segment, with reduced propensity around β2 (Fig. 2a). We next used ZipperDB, which evaluates short hexapeptides for their propensity to form steric-zipper spines (Goldschmidt et al. 2010; Rosenberg et al. 2022). In both WT and AAAA sequences, ZipperDB detected multiple high-scoring windows across the RHIM-containing segment, including regions encompassing the tetrad (Fig. 2b). Together, these analyses suggest that, despite altered side-chain chemistry in the mutant tetrad, the RHIM region retains sequence features compatible with β-aggregation, while different algorithms emphasize different local hotspots.

Finally, we explored structural models with AlphaFold3. For WT, the prediction recapitulated the three-stranded β-sheet scaffold reported for the RIPK3 amyloid core, placing the VQVG tetrad at the center of the fold (Fig. 2c). For the AAAA variant, AlphaFold3 suggested a related β1–β3 topology and a possible dimeric arrangement. However, confidence values were overall lower for the mutant, as reflected by reduced pLDDT scores (Fig. S1), and Alpha-Fold is not designed to robustly predict amyloid architectures. Therefore, these models should be interpreted cautiously and are used here only to illustrate potential packing scenarios involving AAAA vs. VQVG tetrads from β2 (Fig. 2c). Nevertheless, the combined bioinformatic results support the idea that AAAA may retain structural compatibility with a RHIM-like β-architecture, consistent with a latent assembly potential that may be suppressed in bulk by nucleation barriers.

### Anionic membranes enable ThT-positive assembly of WT and the tetrad mutant

Recent studies have shown that membrane interfaces can act as powerful modulators of RIPK3 amyloidogenesis, enabling the wild-type protein to assemble even under conditions that are otherwise non-permissive in bulk solution, such as acidic pH and low ionic strength (Escobedo et al., 2025). Motivated by these findings and by our bioinformatic and structural predictions indicating that the AAAA variant may retain a latent β-aggregation potential (Fig. 2), we next asked whether charged membrane surfaces could unmask this potential and promote nucleation of the mutant. To test this, we monitored ThT fluorescence in the presence of anionic nanodiscs (DMPG DIBMALPs) under acidic, non-permissive conditions where neither WT nor AAAA aggregates in solution.

**Fig. 3:**
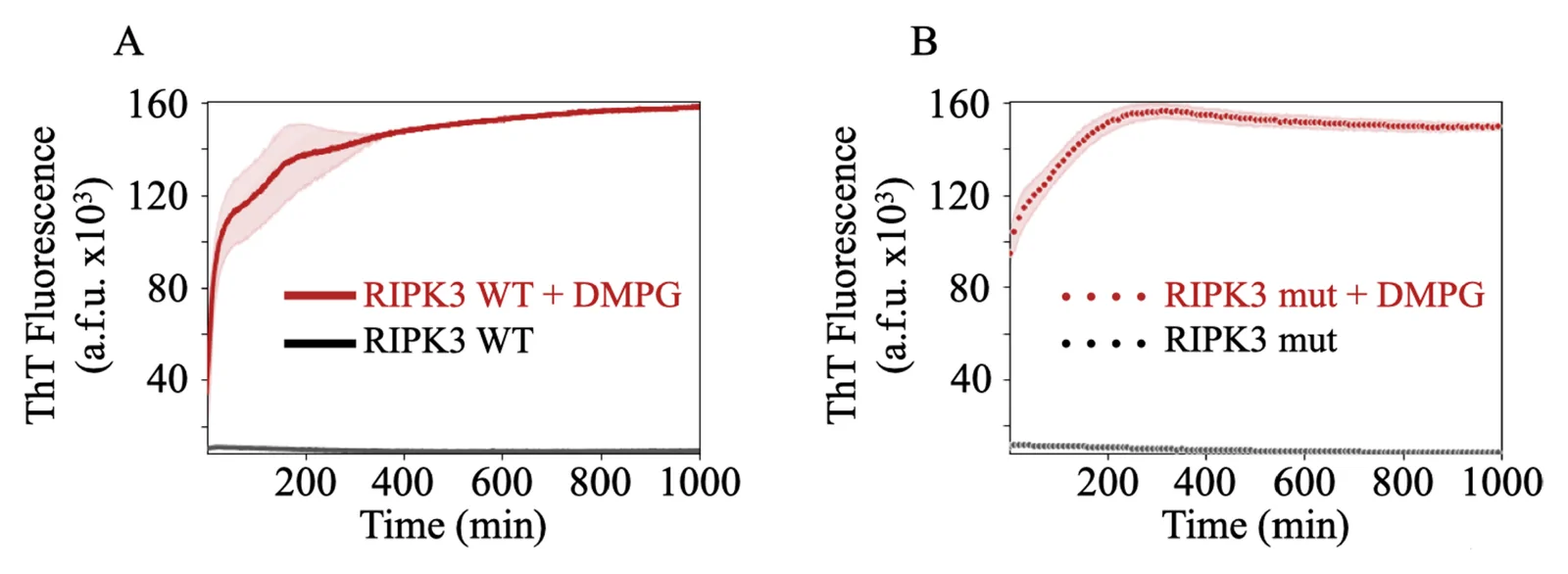
ThT fluorescence assays in the presence of lipid nanodiscs shows that anionic membranes unlock assembly of both RIPK3 WT and the AAAA variant. (**a**) In the presence of neutral nanodiscs (DMPC DIBMALPs), neither WT nor AAAA displays detectable ThT-positive assembly under the conditions tested; (**b**) In the presence of anionic lipidic particles (DMPG DIBMALPs), both WT and AAAA exhibit an increase in ThT fluorescence, consistent with a membrane-enabled assembly pathway

The response was striking. For the WT protein, ThT fluorescence exhibited a rapid and robust rise, consistent with membrane-assisted nucleation followed by amyloid elongation as reported by Escobedo and coworkers (2025) (Fig. 3). Remarkably, the AAAA variant, which was completely inert in bulk solution under any condition tested as shown in Fig. 1, also produced a clear increase in ThT signal upon addition of anionic DIBMALPs. Although the kinetics of fluorescence increase for the AAAA variant appeared slower and the plateau intensity somewhat lower compared to the WT, the overall behavior indicated that the mutant could engage in productive nucleation and assembly when presented with a charged lipid interface.

These results reveal that anionic membranes act as active modulators of RIPK3 amyloidogenesis, concentrating and orienting protein molecules at the bilayer surface in a manner that lowers the kinetic barrier to nucleation. Importantly, this surface-driven pathway does not require the canonical RHIM core interactions that dominate nucleation in bulk solution, thereby exposing a latent structural propensity for amyloid assembly that would otherwise remain silent.

### NMR reveals membrane-dependent recruitment and assembly signatures in the RHIM-disrupted mutant

**Fig. 4:**
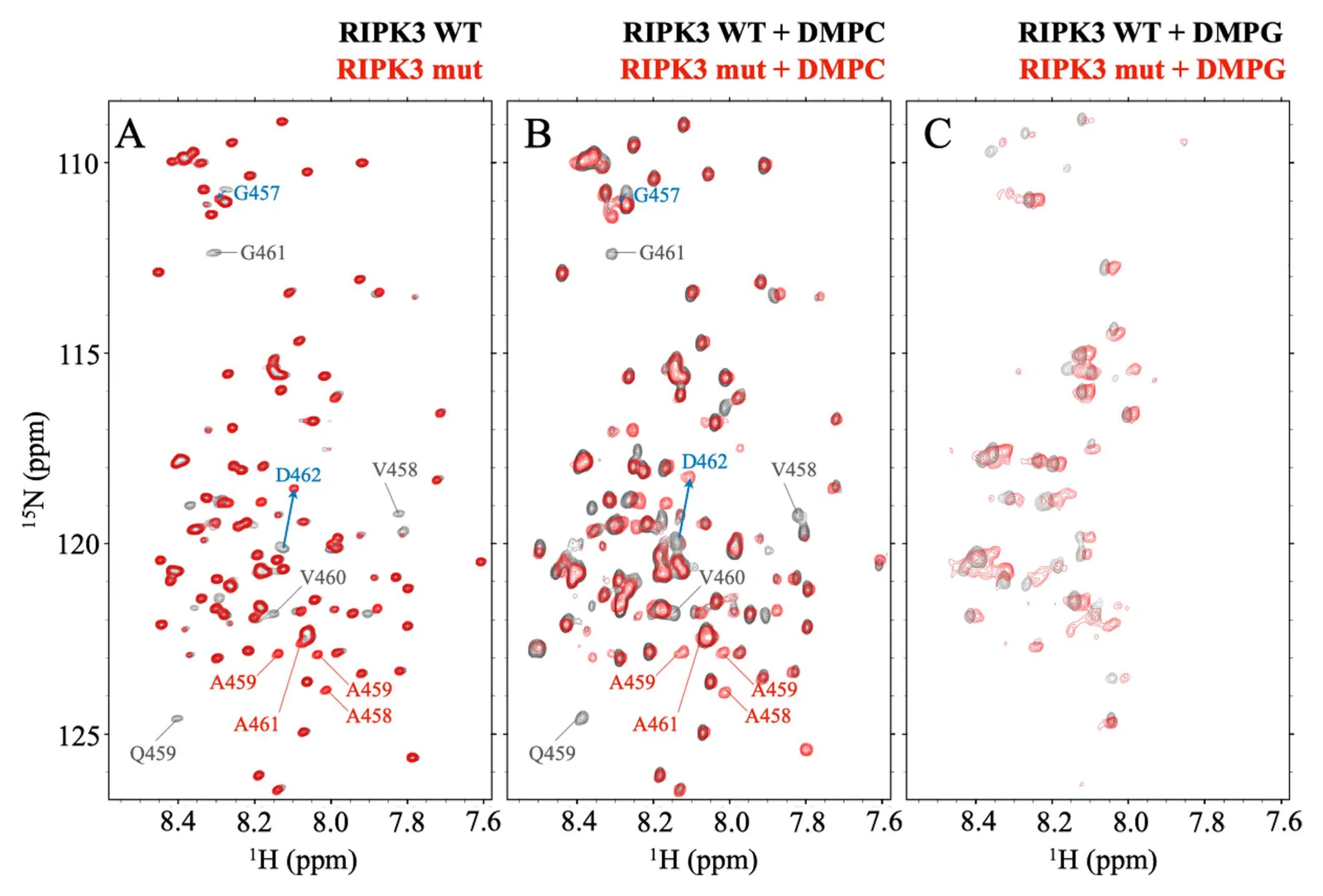
^1^–^15^ N HSQC spectra show comparable behavior of RIPK3 WT and AAAA in the absence and presence of neutral or anionic lipidic particles. (**a**) Overlaid ^1^-^15^ N HSQC spectra of WT (black) and AAAA (red) in the absence of lipid; (**b**) Overlaid spectra after addition of neutral nanodiscs (DMPC DIBMALPs); (**c**) Overlaid spectra after addition of anionic nanodiscs (DMGC DIBMALPs). Resonances assigned to the RHIM tetrad are indicated (VQVG in WT, black; AAAA in the mutant, red). Flanking residues are highlighted in blue, with arrows indicating their chemical shift changes

To gain molecular insight into the membrane-driven pathway uncovered in the ThT assays, we examined the interaction of ^15^N-labeled WT and AAAA variants with nanodiscs using solution ^1^–^15^ N HSQC spectroscopy (Fig. 4). In the absence of lipids, both constructs displayed sharp, well-dispersed resonances across the spectrum, consistent with a monomeric and dynamically disordered state freely tumbling in solution. Addition of neutral DMPC DIBMALPs produced no detectable perturbations in either construct: chemical shifts and line widths remained essentially unchanged, confirming that neutral membranes do not measurably interact with these polypeptides (Fig. 4).

The scenario changed dramatically in the presence of anionic DMPG DIBMALPs. For both WT and AAAA samples, resonances underwent widespread attenuation, with many peaks collapsing into the noise or disappearing entirely. This global signal loss is a well-established signature of a transition to a slower tumbling regime. Consistent with this, the conformational chemical shift profiles of WT and AAAA are highly similar across the two polypeptides (Fig. S2), indicating that the AAAA substitution does not introduce new structured regions or widespread secondary-structure changes in the monomeric state. The similarity of the spectral response between WT and AAAA is striking: despite the lack of aggregation in bulk solution, the AAAA variant is efficiently recruited to the membrane interface and undergoes a comparable transition to a. slow-tumbling regime consistent with early assembly at the membrane surface. This finding provides direct molecular evidence that electrostatic interactions between the negatively charged lipid headgroups and the RIPK3 polypeptides are sufficient to concentrate and immobilize both sequences at the bilayer surface, lowering the kinetic barrier to nucleation.

## Discussion

Our results show that RIPK3 amyloidogenesis is governed by two separable factors: an intrinsic sequence-defined propensity encoded in its RHIM sequence and an environmental component that can modulate nucleation. In bulk solution, the conserved VQVG core tetrad is an indispensable requirement for nucleation, and neither electrostatic screening by NaCl nor changes in pH can overcome this compositional constraint. Yet, bioinformatic and structural models indicate that the substituting the VQVG core tetrad by alanines retains a latent amyloidogenic potential, suggesting that the sequence alone is not incompatible with amyloid formation but that nucleation may be kinetically inaccessible.

Negatively charged membranes unlock this latent potential. By recruiting and locally concentrating RIPK3 segments, they lower the energy barrier for productive intermolecular association and enable a surface-driven nucleation pathway. This is evidenced by both the ThT fluorescence assays and the extensive NMR signal loss, which together reveal strong membrane recruitment and early assembly events for both WT and the AAAA RIPK3 variant. These observations identify the membrane interface as an active modulator, rather than a passive scaffold, of protein amyloidogenesis.

More broadly, our observations align with extensive work on pathological amyloids showing that lipid interfaces can catalyze nucleation by concentrating monomers, restricting conformational freedom, and reshaping the early energy landscape of assembly. In systems such as Tau, Aβ and α-synuclein, membrane charge, composition, and curvature have been shown to modulate oligomer formation and fibril growth, in some cases promoting distinct on-surface pathways relative to bulk solution (Frieg et al. 2024; El Mammeri et al. 2025; Sant et al. 2025). In this context, anionic patches or locally remodeled membranes could act as spatial ‘reaction platforms’ for RIPK3, providing an electrostatically favorable environment that increases local effective concentration and enables productive intermolecular encounters even when bulk nucleation is kinetically hindered.

This dual “sequence and environment” control may have broader implications for the regulation of functional amyloids in cellular contexts. Variations in local lipid composition or charge distribution could act as regulatory switches, tuning the kinetics and spatial localization of RIPK3 assembly during necroptotic signaling. Such spatial and temporal regulation would help ensure that amyloid formation occurs only when and where it is functionally required, while reducing the risk of ectopic or pathological aggregation.

Several questions remain open. Whether membrane-templated assemblies share the same amyloid core and polymorphs as their solution counterparts, or whether they represent distinct structural states constrained by the interface, remains to be determined. Likewise, defining the residues that mediate the initial membrane interaction and resolving the structural organization of AAAA-derived assemblies will be critical to fully delineate the mechanistic basis of this surface-assisted nucleation pathway.

Taken together, these findings provide a conceptual framework in which sequence and environment act as orthogonal determinants of amyloid nucleation. By showing that the latent amyloidogenic potential of RIPK3 can be unmasked by charged membranes, this work highlights the importance of considering both molecular and environmental determinants in the regulation of amyloid assembly.

## Supplementary Material

The online version contains supplementary material available at https://doi.org/10.1007/s00249-026-01833-8.

Supporting Information

## References

[R1] Abramson J, Adler J, Dunger J, Evans R, Green T, Pritzel A, Ronneberger O, Willmore L, Ballard AJ, Bambrick J, Bodenstein SW (2024). Accurate structure prediction of biomolecular interactions with AlphaFold 3. Nature.

[R2] Andrés-Campos S, Titaux-Delgado GA, Escobedo-González FC, Mompeán M (2025). High-quality 13 C-detected structural analysis of mass-limited amyloid samples using a CPMAS CryoProbe and moderate magnetic fields. Solid State Nucl Magn Reson.

[R3] Davies KA, Czabotar PE, Murphy JM (2024). Death at a Funeral: Activation of the Dead Enzyme, MLKL, to Kill Cells by Necroptosis. Curr Opin Struct Biol.

[R4] Dey C, Roy M, Ghosh R, Pal P, Roy D, Ghosh Dey S (2024). Active Site Environment and Reactivity of Copper–Aβ in Membrane Mimetic SDS Micellar Environment. Chem Eur J.

[R5] Dhuriya YK, Sharma D (2018). Necroptosis: A Regulated Inflammatory Mode of Cell Death. J Neuroinflammation.

[R6] El Mammeri N, Duan P, Hong M (2025). Pseudo-Phosphorylated Tau Forms Paired Helical Filaments in the Presence of High-Curvature Cholesterol-Containing Lipid Membranes. J Am Chem Soc.

[R7] Escobedo-González FC, Gelardo A, Reimers A, Polonio P, Mompeán M, Titaux-Delgado GA (2025). Membrane charge primes the necroptotic kinase RIPK3 for amyloid assembly. Commun Chem.

[R8] Frieg B, Han M, Giller K, Dienemann C, Riedel D, Becker S, Andreas LB, Griesinger C, Schröder GF (2024). Cryo-EM Structures of Lipidic Fibrils of Amyloid-β (1–40). Nat Commun.

[R9] Goldschmidt L, Teng PK, Riek R, Eisenberg D (2010). Identifying the amylome, proteins capable of forming amyloid-like fibrils. Proc Natl Acad Sci U S A.

[R10] Lee W, Rahimi M, Lee Y, Chiu A (2021). POKY: a software suite for multidimensional NMR and 3D structure calculation of biomolecules. Bioinformatics.

[R11] LeVine H (1993). Thioflavine T interaction with synthetic Alzheimer’s disease beta-amyloid peptides: detection of amyloid aggregation in solution. Protein Sci.

[R12] Li J, McQuade T, Siemer AB, Napetschnig J, Moriwaki K, Hsiao YS, Damko E, Moquin D, Walz T, McDermott A, Chan FK (2012). The RIP1/RIP3 Necrosome Forms a Functional Amyloid Signaling Complex Required for Programmed Necrosis. Cell.

[R13] Pham CLL, Titaux-Delgado GA, Varghese NR, Polonio P, Wilde KL, Sunde M, Mompeán M (2023). NMR characterization of an assembling RHIM (RIP homotypic interaction motif) amyloid reveals a cryptic region for self-recognition. J Biol Chem.

[R14] Rosenberg GM, Murray KA, Salwinski L, Hughes MP, Abskharon R, Eisenberg DS (2022). Bioinformatic identification of previously unrecognized amyloidogenic proteins. J Biol Chem.

[R15] Samson AL, Murphy JM (2024). Mapping Where and When Necroptotic Cell Death Occurs in Disease. Cell Death Differ.

[R16] Sant V, Matthes D, Mazal H, Antonschmidt L, Wieser F, Movellan KT, Xue K, Nimerovsky E, Stampolaki M, Nathan M, Riedel D (2025). Lipidic Folding Pathway of α-Synuclein via a Toxic Oligomer. Nat Commun.

[R17] Sivashanmugam A (2009). Practical protocols for production of very high yields of recombinant proteins using *Escherichia coli*. Protein Sci.

[R18] He C, Varghese NR, Keeler EG, Pham CLL, Williams B, Tetter S, Semaan C, Wilde KL, Brown SHJ, Bouwer JC, Gambin Y (2025). Structural Studies of an Anti-necroptosis Viral:Human Functional Hetero-amyloid M45:RIPK3 using SSNMR. bioRxiv.

[R19] Walsh I, Seno F, Tosatto SC, Trovato A (2014). PASTA 2.0: an improved server for protein aggregation prediction. Nucleic Acids Res.

[R20] Wu X, Ma Y, Zhao K, Zhang J, Sun Y, Li Y, Dong X, Hu H, Liu J, Wang J, Zhang X (2021). The Structure of a Minimum Amyloid Fibril Core Formed by Necroptosis-Mediating RHIM of Human RIPK3. Proc Natl Acad Sci U S A.

[R21] Ye K, Chen Z, Xu Y (2023). The Double-Edged Functions of Necroptosis. Cell Death Dis.

