# Supplementary material for: Charged membrane interfaces reshape the nucleation landscape of RIPK3 amyloid variants
Source: Eur Biophys J. 2026 Mar 10;55(4):621–9. doi: 10.1007/s00249-026-01833-8 (PMC7618891; doi:10.1007/s00249-026-01833-8)
Supplement: Supplementary file 1 [file 249_2026_1833_MOESM1_ESM.docx]

# *Supporting information for:*

# Charged membrane interfaces reshape the nucleation landscape of RIPK3 amyloid variants

Fátima C. Escobedo-González^1#^, Andrea Gelardo^1#^, Gustavo A. Titaux-Delgado^1^, Miguel Mompeán^1*^,

^1^Instituto de Química Física Blas Cabrera, Consejo Superior de Investigaciones Científicas (IQF-CSIC), Serrano 119, 28006 Madrid, Spain.

#Co-first authors

*Correspondence: [mmompean@iqf.csic.es](mailto:mmompean@iqf.csic.es)

**Figure S1**. **AlphaFold3 confidence metrics for the AAAA RHIM model***.* Per-residue pLDDT values shown as a 1D profile along the sequence. The reduced confidence relative to WT supports interpreting the AAAA model qualitatively and motivates the cautious use of these predictions as illustrative packing scenarios rather than definitive amyloid structures.

**Figure S2. Conformational chemical shift comparison between RIPK3 WT and AAAA CTD constructs***.* Residue-resolved ΔCα–ΔCβ values (WT, black; AAAA, red) derived from newly acquired ^13^C,^15^N backbone assignments under the conditions used for the WT NMR measurements. The highly similar profiles across the CTD indicate comparable secondary-structure propensities in WT and AAAA, with no evidence for widespread long-range structural changes induced by the VQVG→AAAA substitution.
